# Intrastrand backbone-nucleobase interactions stabilize unwound right-handed helical structures of heteroduplexes of L-*a*TNA/RNA and SNA/RNA

**DOI:** 10.1038/s42004-020-00400-2

**Published:** 2020-11-06

**Authors:** Yukiko Kamiya, Tadashi Satoh, Atsuji Kodama, Tatsuya Suzuki, Keiji Murayama, Hiromu Kashida, Susumu Uchiyama, Koichi Kato, Hiroyuki Asanuma

**Affiliations:** 1 Graduate School of Engineering, Nagoya University, Furo-cho, Chikusa-ku, Nagoya, 464-8603 Japan; 2 Graduate School of Pharmaceutical Sciences, Nagoya City University, 3-1 Tanabe-dori, Mizuho-ku, Nagoya, 467-8603 Japan; 3 Exploratory Research Center on Life and Living Systems (ExCELLS), National Institutes of Natural Sciences, 5-1 Higashiyama, Myodaiji, Okazaki, 444-8787 Japan; 4 Institute for Molecular Science, National Institutes of Natural Sciences, 5-1 Higashiyama, Myodaiji, Okazaki, 444-8787 Japan; 5 Graduate School of Engineering, Osaka University, Suita, Osaka 565-0871 Japan

**Keywords:** X-ray crystallography, Nucleic acids

## Abstract

Xeno nucleic acids, which are synthetic analogues of natural nucleic acids, have potential for use in nucleic acid drugs and as orthogonal genetic biopolymers and prebiotic precursors. Although few acyclic nucleic acids can stably bind to RNA and DNA, serinol nucleic acid (SNA) and L-threoninol nucleic acid (L-*a*TNA) stably bind to them. Here we disclose crystal structures of RNA hybridizing with SNA and with L-*a*TNA. The heteroduplexes show unwound right-handed helical structures. Unlike canonical A-type duplexes, the base pairs in the heteroduplexes align perpendicularly to the helical axes, and consequently helical pitches are large. The unwound helical structures originate from interactions between nucleobases and neighbouring backbones of L-*a*TNA and SNA through CH–O bonds. In addition, SNA and L-*a*TNA form a triplex structure via C:G*G parallel Hoogsteen interactions with RNA. The unique structural features of the RNA-recognizing mode of L-*a*TNA and SNA should prove useful in nanotechnology, biotechnology, and basic research into prebiotic chemistry.

## Abstract (web summary)

Serinol nucleic acid and L-threoninol nucleic acid can bind to RNA and DNA, endowing them with potential as nucleic acid-based drugs. Here the authors prepare single crystals of L-*a*TNA/RNA and SNA/RNA heteroduplexes to further our structural understanding of how synthetic nucleic acids hybridize with natural nucleic acids.

## Introduction

Xeno nucleic acids (XNAs) are synthetic analogues that retain natural nucleobases but are replaced with backbone structures different from DNA and RNA. They have potential for use in nucleic acid-based drugs, in development of artificial genetic polymers, and in the prebiotic field^1–8^. In the past decades, many nucleic acid analogues have been developed. Artificial analogues with 2′-ribose modifications such as 2′-fluoro, 2′-*O*-methyl, 2′-*O*-methoxyethyl, and locked nucleic acids (LNAs) are used in the nucleic acid-based drugs and drug candidates^9–14^. The increased binding affinities of these modified analogues for RNA result from the C3′-endo conformation of pentose ring^3,15,16^. Not only RNA analogues alternate sugar-based XNAs have been developed. Six-membered ring-based hexitol nucleic acid (HNA) has restricted ring conformation to form an A-type structure in the duplex with RNA^17,18^. Five-membered ring group α-L-threofuranosyl-(3′→2′) nucleic acid (TNA), which has been used as a model of pre-RNA polymer possesses the phosphodiester group connecting at a different position from that in natural nucleic acids^19–21^. Although the backbone unit of TNA is one atom shorter than that of natural nucleic acids that have six-atom backbone repeat, TNA is capable of forming stable duplex with complementary RNA^19^. In addition to cyclic scaffolds, ribose-inspired acyclic XNAs have been synthesized and characterized because the acyclic XNAs provide extremely resistant against enzymatic degradation^22^. However, acyclic ribose modified analogues decrease the stabilities of the heteroduplex with RNA^23–25^. Also, development of acyclic nucleic acids composing simple structure such as glycerol nucleic acid (GNA), which is most simplified acyclic backbone of propylene glycol^26–28^, and zip nucleic acid, which is connecting through six-bonds system in analogy to natural nucleic acid by phosphonomethylglycerol unit^29^ have been attempted. Although their homoduplexes are highly stable, the stabilities of heteroduplexes with natural nucleic acids are much lower than that of unmodified DNA or RNA duplexes. Peptide nucleic acid (PNA), non-charged type acyclic nucleic acid, binds with high affinity to RNA and DNA^30–32^, however, synthesis of long oligomer and purine-rich sequence is technically very difficult due to poor solubility.

We recently discovered acyclic nucleic acids serinol nucleic acid (SNA) and L-threoninol nucleic acid (L-*a*TNA) can form stable duplexes with RNA in a sequence-specific manner^33–35^. Since they are structurally simple, readily synthesized, excellent water solubility, and high nucleases resistance, various applications have been realized based on hybridization with RNA such as a high-sensitive molecular beacon and nucleic acid-based drug candidates, including siRNAs, anti-miRNA oligonucleotides, and exon-skipping type antisense oligonucleotides^36–43^. However, how SNA and L-*a*TNA hybridize with natural nucleic acid have remained unknown. We initially assumed that helicity of duplex of D-*a*TNA, which is enantiomer of L-*a*TNA, is right handed based on the similarity of CD spectrum to that of DNA and structural modelling^34,44^, and that L-*a*TNA homoduplex had left-handed helicity. However, based on CD studies of PNA of known structure^45^, the CD signal of L-*a*TNA is indicative of right-handed helicity^35^. Interestingly, the handedness of an SNA homoduplex depends on the oligomer sequence^33^. Despite the importance of the acyclic nucleic acid, only limited structural information is available. For development of acyclic nucleic acids, for applications to tools or materials, and to facilitate de novo design of artificial polymerases for these acyclic nucleic acids, structural information is helpful. In the present study, we successfully prepared single crystals of L-*a*TNA/RNA and SNA/RNA heteroduplexes and solved the first crystal structures at 1.70–1.75 Å resolution. L-*a*TNA/RNA and SNA/RNA form right-handed helical structures with large helical pitch involving Watson–Crick base pairs and parallel-type Hoogsteen base pairs.

## Results

### Helicities and paring mode of L-*a*TNA/RNA and SNA/RNA

The duplexes formed by an L-*a*TNA strand _L_T8a (3′-GCAGCAGC-1′) with an RNA strand R8Br (5′-GCUGC-_Br_U-GC-3′) and an SNA strand S8a ((*S*)-GCAGCAGC-(*R*)) with the R8Br were prepared. Melting analyses and CD spectroscopy analyses confirmed that complexes were formed (Supplementary Fig. 1). Crystals were obtained using the sitting drop vapour diffusion method with PEG400 as precipitant. The L-*a*TNA/RNA crystal diffracted at 1.5-Å resolution and the SNA/RNA crystal diffracted at 1.7-Å resolution. Data collection and structure refinement statistics are summarized in Tables 1 and 2. The structures were solved by X-ray anomalous scattering using Br atom of _Br_U on RNA strand.

**Table 1:** Data collection, phasing, and refinement statistics for L-*a*TNA/RNA.

	Native		MAD dataset	
**Data collection**				
Space group	*I*2	*C*2	*C*2	*C*2
Cell dimensions				
*a*, *b*, *c* (Å)	53.0, 33.3, 54.1	57.7, 33.6, 53.4	57.7, 33.6, 53.4	57.8, 33.6, 53.4
α, β, γ (°)	90.0, 115.7, 90.0	90.0, 122.1, 90.0	90.0, 122.1, 90.0	90.0, 122.1, 90.0
		*Peak*	*Inflection*	*Remote*
Wavelength	0.9000	0.9197	0.9200	0.9000
Resolution (Å)	28.50–1.75	50.00–1.50	50.00–1.50	50.00–1.50
	(1.78–1.75)	(1.59–1.50)	(1.59–1.50)	(1.59–1.50)
*R*_merge_	6.0 (30.7)	4.2 (30.1)	3.9 (24.4)	6.5 (27.0)
*I*/σ*I*	13.9 (4.6)	8.1 (1.0)	9.0 (1.2)	7.8 (1.3)
Completeness (%)	98.7 (99.1)	91.3 (92.0)	91.1 (92.3)	93.3 (93.4)
Redundancy	4.3 (4.5)	1.9 (1.9)	1.9 (1.9)	2.4 (2.4)
**Refinement**				
Resolution (Å)	20.0–1.75	–	–	–
No. reflections	8218	–	–	–
*R*_work_/*R*_free_	26.9/28.8	–	–	–
No. atoms				
RNA (A/B)	147/147	–	–	–
L-*a*TNA (C/D)	178/178	–	–	–
Water	21	–	–	–
*B*-factors				
RNA (A/B)	55.2/49.3	–	–	–
L-*a*TNA (C/D)	45.8/43.7	–	–	–
Water	44.9	–	–	–
R.m.s deviations				
Bond lengths (Å)	0.007	–	–	–
Bond angles (°)	1.66	–	–	–

**Table 2:** Data collection, phasing, and refinement statistics for SNA/RNA.

	Native and MAD dataset	
**Data collection**		
Space group	*P*2_1_	*P*2_1_
Cell dimensions		
*a*, *b*, *c* (Å)	40.4, 46.9, 47.5	40.3, 47.1, 47.5
α, β, γ (°)	90.0, 114.5, 90.0	90.0, 114.5, 90.0
	*Peak*	*Inflection*
Wavelength	0.9197	0.9201
Resolution (Å)	46.92–1.70	47.10–1.75
	(1.73–1.70)	(1.78–1.75)
*R*_merge_	5.2 (97.0)	5.4 (110.7)
*I*/σ*I*	14.3 (2.0)	11.7 (1.0)
Completeness (%)	99.8 (98.5)	99.7 (96.4)
Redundancy	6.9 (6.9)	6.9 (6.7)
**Refinement**		
Resolution (Å)	20.0–1.70	–
No. reflections	16997	–
*R*_work_/*R*_free_	23.1/27.1	–
No. atoms		
RNA (A/C/E/G)	167/167/167/147	–
SNA (B/D/F/H)	170/170/170/170	–
Water	66	–
*B*-factors		
RNA (A/C/E/G)	67.3/66.7/72.8/56.4	–
SNA (B/D/F/H)	55.4/56.2/52.8/57.8	–
Water	54.6	–
R.m.s deviations		
Bond lengths (Å)	0.010	–
Bond angles (°)	1.57	–

**Fig. 1:**
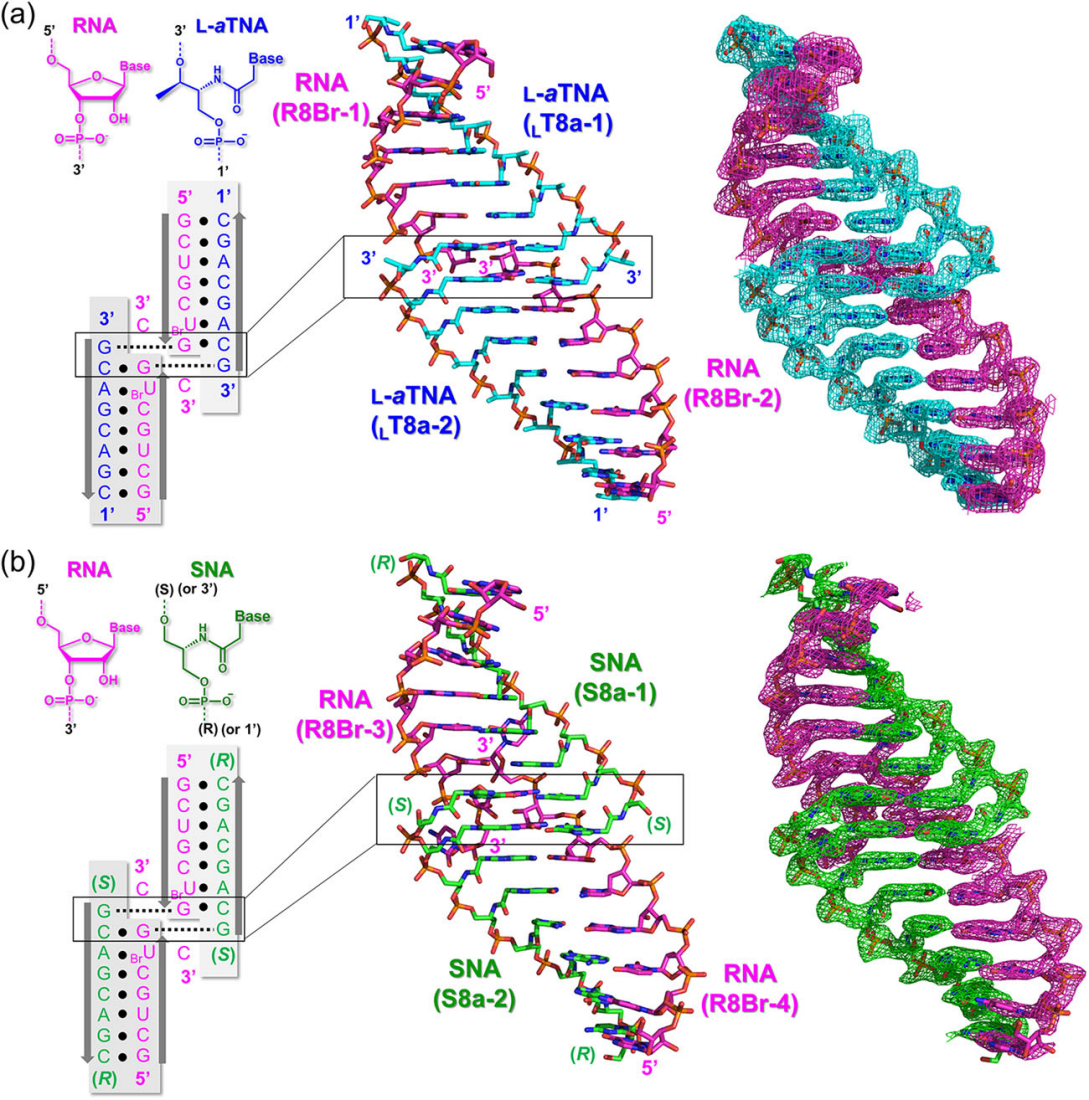
Dimer of duplex structures of L-*a*TNA/RNA and SNA/RNA stabilized by Hoogsteen base pairs. Base-pairing patterns and structures of (**a**) L-*a*TNA/RNA and (**b**) SNA/RNA. In the sequence schematics, Watson–Crick and Hoogsteen base pairs are indicated as black circles and dashed lines, respectively. In the stick representations, carbon atoms are coloured magenta, cyan, and green, nitrogens are in blue, oxygens are in red, and phosphorus is in orange. 2*F*_*o*_*-F*_*c*_ electron density maps contoured at 1.0 σ level. The structures were deposited in the Protein Data Bank. PDB accession codes: 7BPF (L-*a*TNA/RNA) and 7BPG (SNA/RNA).

**Fig. 2:**
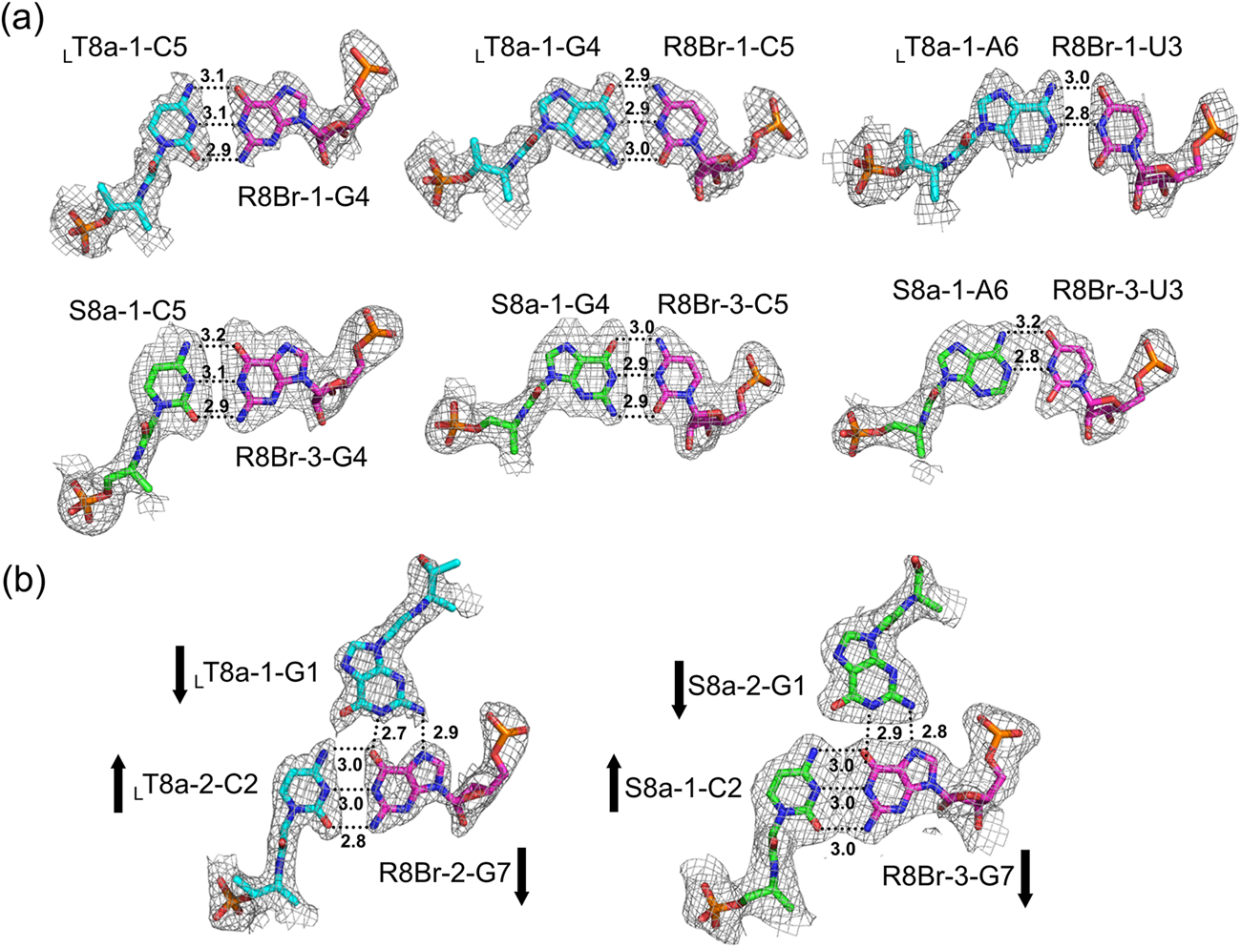
Canonical Watson–Crick and parallel-type Hoogsteen base pairing patterns of L-*a*TNA/RNA and SNA/RNA. Watson–Crick (**a**) and Hoogsteen (**b**) interactions in _L_T8a/R8Br and S8a/R8Br. C, N, O, and P atoms are coloured as in Fig. 1. 2*F*_*o*_*-F*_*c*_ electron density maps contoured at 1.0 σ level. Arrows indicate relative polarities of the backbones of RNA, L-*a*TNA, and SNA.

**Fig. 3:**
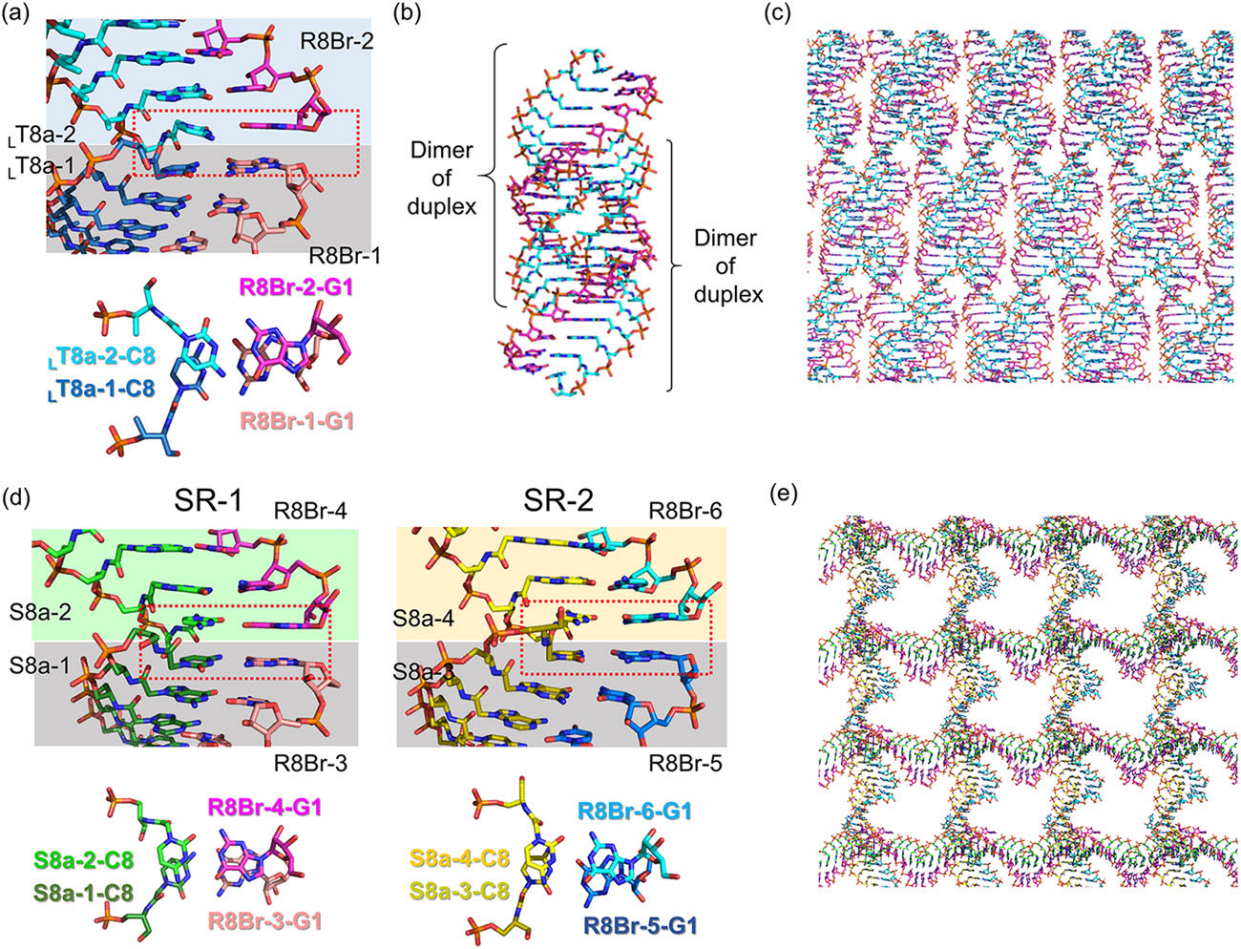
Dimers of L-*a*TNA/RNA and of SNA/RNA duplexes connected by end-to-end stacking pack together in crystals. **a** Stick representations showing end-to-end stacking of two L-*a*TNA/RNA duplexes. Top view of the end-to-end stacking between terminal base pairs is also indicated. **b** High-order helical structure of two dimers of the L-*a*TNA/RNA duplex. **c** Packing observed in the crystal of L-*a*TNA/RNA. **d** Stick representations showing end-to-end stacking of SR-1 and SR-2 SNA/RNA duplex. Top view of the end-to-end stacking between terminal base pairs is also indicated. **e** Packing observed in the crystal of SNA/RNA. Carbon atoms are coloured magenta, pink, cyan, sky blue, green, dark green, yellow, and olive. N, O, and P atoms are coloured as in Fig. 1.

In the L-*a*TNA/RNA heteroduplex structure, the crystallographic asymmetric unit contains two right-handed duplexes (_L_T8a-1/R8Br-1 and _L_T8a-2/R8Br-2) stabilized through canonical Watson–Crick base pairing (Fig. 1a). We refer to the duplex orientation as antiparallel in analogy to natural dsRNA. Hydrogen-bonding distances of Watson–Crick base pairs of G:C and A:U are consistent with those observed in RNA/RNA duplexes (Fig. 2). The two L-*a*TNA/RNA duplexes in the asymmetric unit are surprisingly connected via triplex interactions: C2(_L_T8a-1):G7(R8Br-1)*G1(_L_T8a-2) and C2(_L_T8a-2):G7(R8Br-2)*G1(_L_T8a-1) (the colons indicate the Watson–Crick pairs and the asterisks indicate Hoogsteen interactions) (Figs. 1a and 2b). The electron densities of the 3′-terminal C8s of R8Br-1 and R8Br-2 were not observed, suggesting that these bases were flipped out from the helical structure (Supplementary Fig. 2). This is likely necessary to allow Hoogsteen base pairing for triplex formation. The direction of Hoogsteen base pairing of the G*G interactions in the L-*a*TNA/RNA structure is more similar to that observed in the G-quadruplex structure than in the conventional triplex^46,47^. The L-*a*TNA and L-*a*TNA strands are antiparallel in the triplex region, therefore, we refer to Hoogsteen base pairing of L-*a*TNA and RNA strands as parallel. Additionally, high-order helical structure also stabilizes the crystal of the L-*a*TNA/RNA heteroduplex (Fig. 3a). The two dimers of heteroduplexes connected by the triplex interactions stack end-to-end into a continuous helix. In addition, the dimer of heteroduplexes is wrapped with another dimer of duplexes structure in an antiparallel direction, and these helical structures are tightly packed in the crystal lattice (Fig. 3b, c).

The crystallographic asymmetric unit of SNA/RNA contains four SNA strands (S8a-1 to 4) and four RNA strands (R8Br-3 to 6) (Fig. 1b and Supplementary Fig. 3). Similar to the L-*a*TNA/RNA duplex, a single SNA/RNA duplex is an antiparallel right-handed helix stabilized by canonical Watson–Crick base pairing, and two duplexes are stabilized by formation of two parallel triplex through Hoogsteen base pairs of C(SNA):G(RNA)*G(SNA), result in formation of dimer of duplex structure (Fig. 2). Although electron densities of the C8 residues at 3′-terminus of RNA strands are not observed in the crystal structure of L-*a*TNA/RNA, flipped-out C8s of RNA strands (R8Br-3, -4, and -6) are observed in the SNA/RNA crystal (Supplementary Fig. 4). As in the L-*a*TNA/SNA crystal, the dimers of SNA/RNA duplex are stacked in an end-to-end manner, thereby forming continuous helices in the both perpendicular and horizontal directions (Fig. 3d, e).

### Unwound helical structures of L-*a*TNA/RNA and SNA/RNA duplexes

**Fig. 4:**
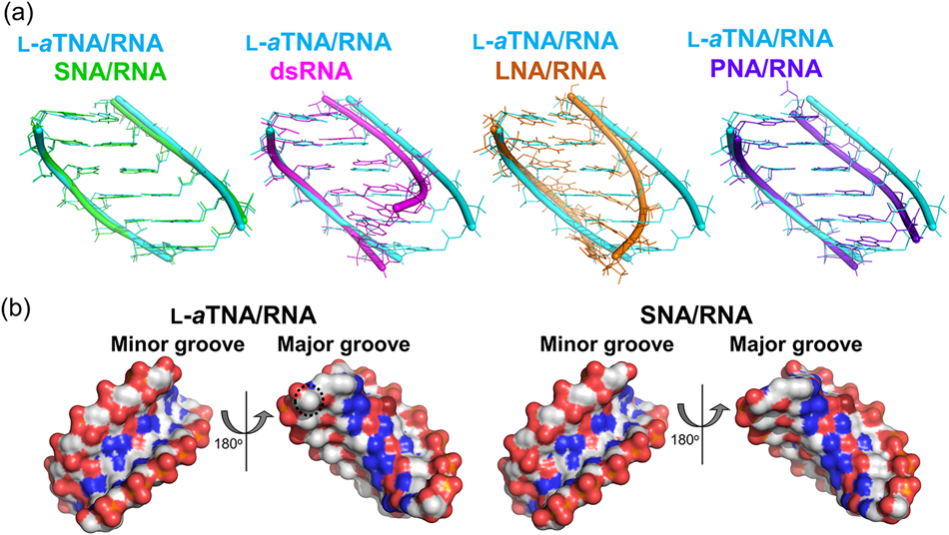
Structures of L-*a*TNA/RNA and SNA/RNA are unwound in comparison to A-type duplex structure. **a** Superposition of duplex structures of L-*a*TNA/RNA (cyan) and SNA/RNA (green), dsRNA (magenta), LNA/RNA (orange), or PNA/RNA (purple) are shown. PDB accession codes were the following: RNA/RNA, 3ND4; LNA/RNA, 1H0Q; PNA/RNA, 5EMF. **b** Surface views of minor and major groove of L-*a*TNA/RNA and SNA/RNA. An example of location of CH_3_ on L-*a*TNA backbone is shown as dotted circle. Carbon atoms are coloured white and N, O, and P atoms are coloured as in Fig. 1.

The helical parameters of the Watson–Crick duplex regions of L-*a*TNA/RNA and SNA/RNA structures were calculated using the program 3DNA (Table 3 and Supplementary Tables 1 and 2)^48–50^. Sugar puckers of most RNA residues are C3′-*endo*, categorized in *Northern*-type, that observed in a typical A-type duplex. Sugars of G1 of R8Br-1 and 2 in the L-*a*TNA/RNA duplexes and sugars of G1, C2 of R8Br-3, C2, G4, and _Br_U6 of R8Br-4, and G1 of R8Br-6 in the SNA/RNA duplexes adopt the C2′-*exo* conformation that lies in the *Northern* range. However, interestingly, helical structures of L-*a*TNA/RNA and SNA/RNA are apparently different from those of RNA/RNA and LNA/RNA which structures are typically categorized in A-type duplex (Fig. 4a). The helical axis-base inclinations for L-*a*TNA/RNA (average 1.6°) and SNA/RNA (average 1.2°) are much smaller than that of A-type RNA/RNA duplex (average 16.6° calculated from PDB 3ND4) and other XNA/RNA heteroduplexes (Table 3)^51^. The values are similar to that observed in B-type DNA duplexes (average 4.4° observed in PDB 3BSE), in which base pairs are stacked in perpendicular to the axis^52^. In contrast, values of X-displacement of L-*a*TNA/RNA and SNA/RNA are much higher than that of dsDNA which show no displacement from the helical axis (−6.4 Å and −5.7 Å for L-*a*TNA/RNA and SNA/RNA, −0.5 Å for dsDNA). The lower inclination and larger displacement cause lower helical twist of the L-*a*TNA/RNA and SNA/RNA structures (22.7° and 24.2° for L-*a*TNA/RNA and SNA/RNA, respectively) compared to 33.0° for dsRNA and 30.0° for LNA/RNA (Table 3). The duplex structures of L-*a*TNA/RNA and SNA/RNA look similar to that of PNA/RNA duplex (RMSD 1.1 Å between N2′, C7′, and C8′ of PNA, and C1′ and P of RNA in PNA/RNA duplex and N4′, C5′, and C6′ of L-*a*TNA, and C1′ and P of RNA in L-*a*TNA/RNA duplex) (Fig. 4a). However, the base pairs in the PNA/RNA duplex appear to be tilted rather than parallel although base pairs of L-*a*TNA/RNA are kept parallel one another (Supplementary Fig. 5)^32^. Due to this, position of helical axis and the value of helical parameters are different from those of L-*a*TNA/RNA and SNA/RNA (Table 3). Thus L-*a*TNA/RNA and SNA/RNA duplexes form unwound and stretched structures with wider major grooves and minor grooves relative to dsRNA and dsDNA (Fig. 4b and Table 3)^16,18,24^.

**Table 3:** Average helical parameters of XNA/RNA, RNA/RNA, and DNA/DNA duplexes^a^.

	L-*a*TNA/RNA	SNA/RNA	LNA/RNA	PNA/RNA	dsRNA	dsDNA
X-displacement [Å]^b^	−6.4	−5.7	−5.4	−6.2	−4.2	−0.5
Inclination [deg]^b^	1.6	1.2	11.6	11.5	16.6	4.4
Helical rise [Å]^b^	3.1	3.2	2.6	2.8	2.7	3.3
Helical twist [deg]^b^	22.7	24.2	30.0	25.7	33.0	34.0
Residues per turn [nt]	15.8	15.3	12.0	14.1	10.9	10.6
Helical pitch [Å]	49.5	47.6	31.0	39.1	28.9	34.6
Minor groove [Å]^c^	9.5	9.4	9.9	n.d.^d^	10.1	5.8

^a^All parameters were calculated using 3DNA-Web. PDB accession codes were the following: LNA/RNA, 1H0Q; PNA/RNA, 5EMF; dsRNA, 3ND4; and dsDNA, 3BSE.

^b^Local base-pair helical parameters.

^c^Minor groove widths are measured as the closest interstrand P–P distances subtracted by 5.8 Å to account for the van der Waals radii of the phosphate groups.

^d^Distances cannot be measured due to double conformation.

### Torsion angles of RNA, L-*a*TNA, and SNA

**Fig. 5:**
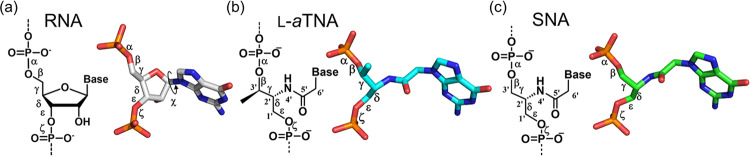
Comparison of backbone torsion angles of RNA, L-*a*TNA, and SNA. **a**–**c** Chemical structures of nucleotides and stick representations in the context of a duplex with RNA for RNA (**a**), L-*a*TNA (**b**), and SNA (**c**). Carbon atoms are shown in white, cyan, and green for RNA, L-*a*TNA, and SNA, respectively. N, O, and P atoms are coloured as in Fig. 1.

To see how RNAs in L-*a*TNA/RNA and SNA/RNA adapt unwound helical structures, we next compared torsion angles of RNAs in dsRNA, L-*a*TNA/RNA, and SNA/RNA duplexes (Fig. 5, Table 4, and Supplementary Tables 3 and 4). The values for the backbone torsion angle **δ** associated with the sugar ring (C5′-C4′-C3′-O3′) of the RNA in the heteroduplex with L-*a*TNA and SNA are between 70° and 100°, the range observed for typical A-type helix^3^, with the exception of those for the terminal residues of each strand. In contrast, the average values for the backbone torsion angle **α** associated with the phosphodiester bond (O3′-P-O5′-C5′) are relatively smaller for the RNA strand in the heteroduplexes than those observed in the dsRNA duplex. The average values for the glycosidic torsion angle **χ** between sugar and base (O4′-C1′-N1-C2 for pyrimidines, O4′-C1′-N9-C4 for purines) are also smaller than those observed in the dsRNA. Differences of these angles, although which are very little, make P–P distances of the L-*a*TNA/RNA and SNA/RNA larger. Consequently, helical pitches of the heteroduplexes larger relative to that of the dsRNA (Table 4).

**Table 4:** Average torsion angles and phosphorous-phosphorous distances for RNA strand in dsRNA, L-*a*TNA/RNA, and SNA/RNA duplexes.

Duplex	α [deg]	β [deg]	γ [deg]	δ [deg]	ε [deg]	ζ [deg]	χ [deg]	P–P [Å]
dsRNA	−66.1	172.0	63.9	78.7	−149.7	−72.0	−160.8	5.8
	(4.6)	(4.7)	(30.7)	(2.6)	(5.5)	(5.7)	(4.1)	(0.3)
L-*a*TNA/RNA	−75.4	173.3	53.1	78.4	−153.8	−75.6	−171.4	6.0
	(22.9)	(10.2)	(47.6)	(7.4)	(7.8)	(8.5)	(5.1)	(0.3)
SNA/RNA	−95.0	162.0	100.2	81.9	−149.5	−99.9	−171.7	6.1
	(53.8)	(28.9)	(71.8)	(9.5)	(20.2)	(73.5)	(7.9)	(0.4)

Average values were calculated from the duplex regions of crystal structures of dsRNA, L-*a*TNA/RNA (G1 ~ _Br_U6), and SNA/RNA (G1 ~ _Br_U6).

While torsion angles of L-*a*TNA and SNA are also defined as **α** to **ζ** (Fig. 5), the number of bonds between the backbone to nucleobase N1 of pyrimidines or N9 of purines are different: three bonds from the C4′ to N1 or to N9 linkage in RNA, whereas 4 bonds from C2′ (corresponding to C4′ of ribose) to N1 or to N9 in the L-*a*TNA and SNA. Additionally, there exist the amide group connecting between backbone-nucleobase on L-*a*TNA and SNA backbones. As expected from the chemical structure differences between L-*a*TNA or SNA and RNA, the torsion angles **α** to **ζ** of L-*a*TNA and SNA are different from those observed in RNA (Fig. 5 and Table 5).

**Table 5:** Average torsion angles and phosphorous-phosphorous distances of L-*a*TNA and SNA strands in L-*a*TNA/RNA and SNA/RNA duplexes.

Duplex	α [deg]	β [deg]	γ [deg]	δ [deg]	ε [deg]	ζ [deg]	P–P [Å]
**L-*a*TNA/RNA**	87.7	173.0	−55.9	−64.8	−138.2	59.1	6.1
	(7.4)	(6.5)	(4.5)	(5.3)	(7.6)	(11.1)	(0.4)
**SNA/RNA**	77.8	−172.6	−58.6	−60.3	−151.0	69.5	6.0
	(15.1)	(8.1)	(5.3)	(5.8)	(10.3)	(9.4)	(0.1)

Average values were calculated from the duplex regions of crystal structures of L-*a*TNA/RNA (C2 ~ G7) and SNA/RNA (C2 ~ G7).

### Intrastrand interactions stabilize L-*a*TNA or SNA in helical structures

**Fig. 6:**
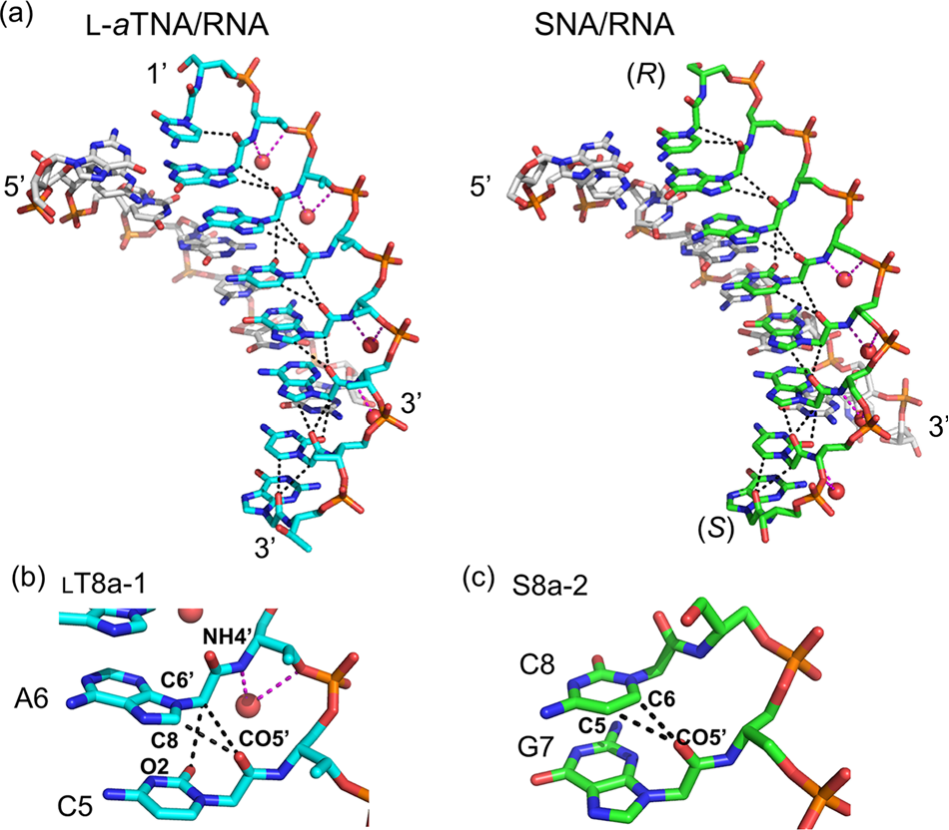
Intrastrand hydrogen bonds between backbone carbonyl oxygens and neighbouring nucleobases. **a** Short C–O distance within the van der Waals distance cutoff of 3.7 Å observed in L-*a*TNA/RNA and SNA/RNA are indicated as dashed line in black. Hydrogen bonds between backbone and water are also indicated as dashed line in magenta. **b** Close up view of C–O interactions of O5′ (backbone)–C8 (adenine), O5'–C6' (backbone), and O2 (cytosine)–C6′ and hydrogen bonds of amide (backbone)–water molecule and O3′–water molecule. **c** Close up view of C–O interactions of O5′–C6 (cytosine) and O5′–C5 (cytosine) at terminal cytosine residue in SNA. Carbon atoms are shown in white, cyan, and green for RNA, L-*a*TNA and SNA, respectively. N, O, and P atoms are coloured as in Fig. 1.

Nucleobases of L-*a*TNA and SNA are connected to backbone through amide bond. All amide bonds in the backbones of L-*a*TNA and SNA are in *trans* configuration and the carbonyl oxygens are turned inward in helical structures of L-*a*TNA/RNA and SNA/RNA (Fig. 6). Parts of NH of L-*a*TNA and SNA backbone form water-mediated hydrogen bonds to O3′ of phosphodiester linkage (Fig. 6). Interestingly the carbonyl oxygens are located in close proximity to the C8 atoms of guanine/adenine or C6 atoms of cytosine of residues next to 1′-terminal (C–O distance: 3.0–4.0 Å) and C6′ atoms of backbone of residues next to 1′-terminal (C–O distance: 3.2–5.0 Å) (Fig. 6 and Supplementary Table 5). Except for the terminal residue most of C–O distances are within the van der Waals distance cutoff of 3.7 Å. In SNA case, C5 atom of terminal cytosine residues instead of C6′ atom of backbone are adjacent to carbonyl oxygen of neighbouring guanine residue (C–O distance: 3.2–3.5 Å). In addition, C–O distances between carbonyl oxygen at C2 position of cytosine and C6′ of neighbouring residues are located closely (3.1–3.4 Å). These observations suggest that weak CH–O hydrogen bonds between nucleobase and backbone of neighbouring residues are extensively formed in intrastrand of L-*a*TNA and SNA (Fig. 6). C8 of purine and C5 and C6 of pyrimidine in aromatic are relatively polarized, therefore carbonyl oxygens putatively act as an acceptor of CH–O hydrogen bonds^53^. The CH–O interactions between aromatic CH and carbonyl oxygens are observed in PNA of PNA/RNA heteroduplex in which PNA backbone and nucleobases are connected by amide bonds^32^. Tendency of CH–O interactions is lower than those observed in L-*a*TNA/RNA and SNA/RNA duplexes, therefore helicity of PNA/RNA duplex is increased in comparison to L-*a*TNA/RNA and SNA/RNA, even it is lower than the case of dsRNA (Table 3). In addition, unsatisfied number of CH–O interactions might cause backbone flexibility of PNA/RNA duplex. These findings reveal that intrastrand hydrogen bonding networks between nucleobases and neighbouring backbones of L-*a*TNA and SNA enable stabilization of acyclic structures and adjust formation of heteroduplexes with RNA.

### Dimer formations of L-*a*TNA/RNA and SNA/RNA duplexes in solution

**Fig. 7:**
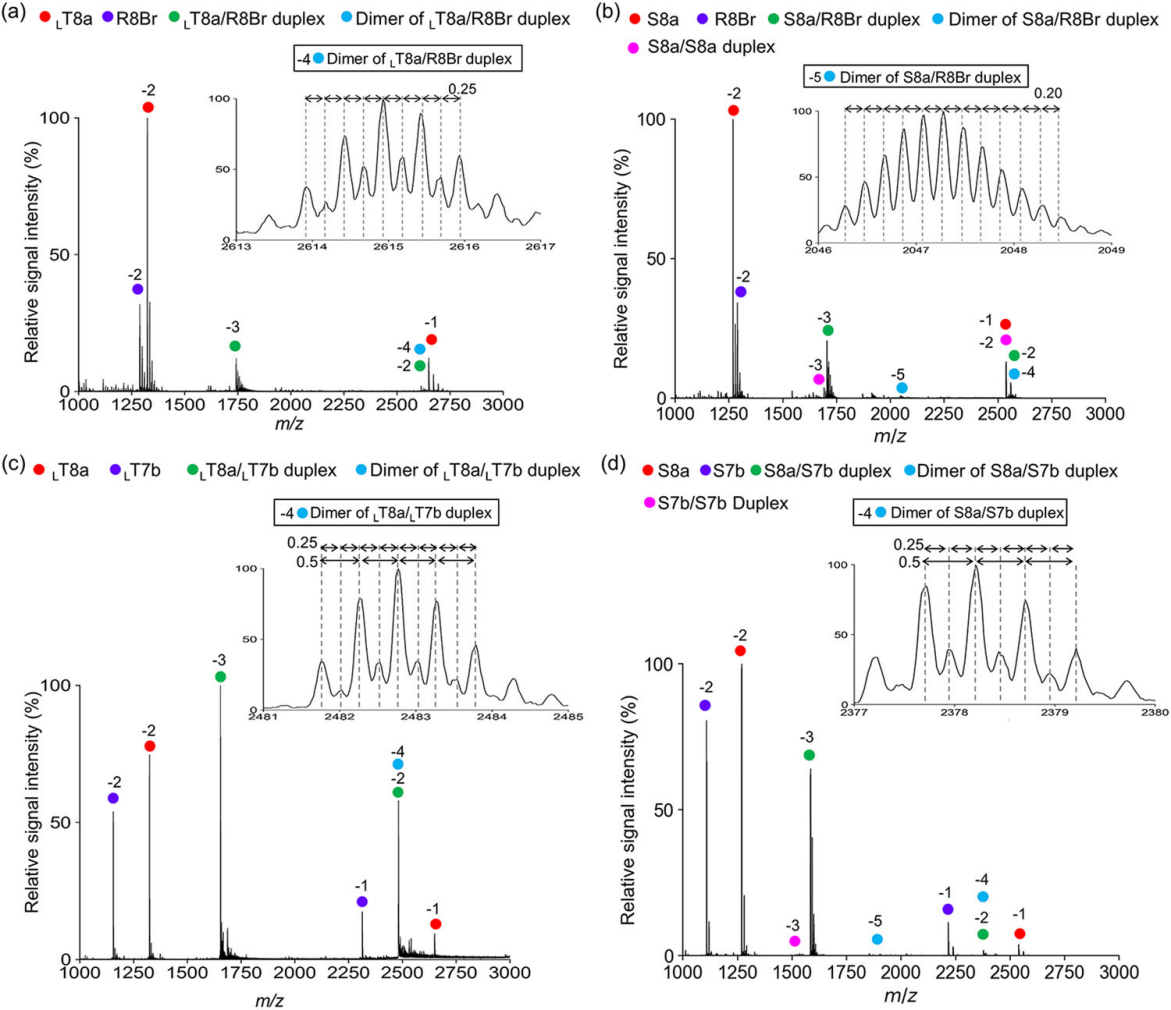
Dimer formation of L-*a*TNA/RNA, SNA/RNA, L-*a*TNA/ L-*a*TNA, and SNA/SNA duplexes in solution. Mass spectrum of solution of _L_T8a and R8Br (**a**), S8a and R8Br (**b**), _L_T8a and _L_T7b (**c**), and S8a and S7b (**d**) were collected under non-denaturing conditions in negative ionization mode. Calculated masses are summarized in materials and methods.

Finally, in order to investigate whether the C:G*G triplex-mediated dimer of duplex structures are formed between XNA and RNA in solution, we performed nanoESI-MS analyses of solutions of _L_T8a and R8Br and of S8a and R8Br under non-denaturing conditions. In the spectrum of the solution of _L_T8a and R8Br, a peak at the expected molecular mass of the dimer of heteroduplexes was observed as were peaks corresponding to single strands and the _L_T8a/R8Br duplex (Fig. 7a). A peak of the expected mass of the dimer of S8a/R8Br heteroduplexes was also observed (Fig. 7b). These results strongly suggest that the C:G*G triplex-stabilized dimers of duplexes form in solution. Then we are interested in whether the triplex are formed by homooligomers. nanoESI-MS analyses of mixtures of the 8-mer GCAGCAGC and the 7-mer GCU(T)GCU(T)G of each type of oligomer were performed. In case of RNA, peaks were observed in the spectrum corresponding to the single strands of R8a and R7b and the duplex of R8a/R7b, but peak corresponding to the dimer of R8a/R7b duplex was not (Supplementary Fig. 6). For L-*a*TNA, which forms stable homoduplex structure, the peak of dimers of _L_T8a/_L_T7b duplex was clearly observed (Fig. 7c and Supplementary Fig. 7). The same result was obtained from the S8a and S7b case (Fig. 7d). These data suggest that homo-L-*a*TNA and homo-SNA oligomers can form parallel-type Hoogsteen base pairs and the Hoogsteen base triplex interactions uniquely stabilize formation of dimers of L-*a*TNA and SNA duplexes.

## Discussion

Crystal structures of L-*a*TNA/RNA and SNA/RNA clearly show L-*a*TNA and SNA form right-handed duplex structures with RNA in a Watson–Crick base pairing. Although the sugar puckering of RNA in these duplexes are *N*-type configuration which usually observed in A-type duplex form, the helical twists are smaller, the rises are larger, and inclinations are smaller in comparison to the dsRNA and the duplexes of RNA with 2′-modified RNA or LNA. The geometries of L-*a*TNA/RNA and SNA/RNA duplexes have greater helical pitches and base pairs aligned parallel to the helical axes. Carbonyl oxygens of amide bonds connecting between nucleobases and backbones interact with neighbouring nucleobases via CH–O hydrogen bonds. This is not contained in natural nucleic acid. We suggest that CH–O hydrogen bonds in addition to increases of π-π interactions within the stacked nucleobases stabilize unwound helical structures of L-*a*TNA and SNA in heteroduplexes with RNA. CH–O hydrogen interactions are known to be involved in folding of peptides, proteins, and tRNA, and also molecular-molecular interactions^54–57^. It is possible that artificial nucleic acid having desired helicity and folding can be designed by introduction of CH–O hydrogen bonds at certain point.

In the previous work, we demonstrated that D-*a*TNA does not form stable duplexes with RNA^34^. The difference among the D-*a*TNA, L-aTNA, and SNA monomer is presence or position of single methyl group. It is likely that the methyl group in D-*a*TNA induces steric crash with oxygens of neighbouring phosphate group if it is right-handed helical structure (Supplementary Fig. 8). Also, it is possible that methyl group, which is located in major groove in the L-*a*TNA/RNA duplex but in minor groove in the putative right-handed D-*a*TNA/RNA duplex, has a negative effect on minor groove environment (Fig. 4b and Supplementary Fig. 8). To avoid them, D-*a*TNA is expected to prefer left-handed helix formation.

We found that two duplexes interact through triplex formations to form dimer of duplex structures. Interestingly the triplexes were formed by parallel-type Hoogsteen base pairing between Gs and a Watson–Crick G:C base pairs. Conventionally G:C Watson–Crick base pair in DNA and RNA forms parallel Hoogsteen base pair with C, but this type of interaction is disfavoured at physiological pH because protonation of C in third strand is required for formation of the Hoogsteen interaction^58–60^. In addition, canonical C:G*G type triplex is formed by antiparallel direction between G and G^60^. In the L-*a*TNA/RNA and SNA/RNA, the orientations of the third nucleobase (G) are different from that observed in typical C:G*G triplex. This allows them to form parallel type Hoogsteen interaction. The unique triplex forming abilities of L-*a*TNA and SNA will be an advantage for applying triplex-based theranostics.

Nucleic acids play important roles as the blueprints for construction of all living organisms. It is hypothesized that RNA served as the precursor in a prebiotic world^6–8,21^. On the other hand, acyclic type XNA studies showed that acyclic nucleic acids can form homoduplex and heteroduplex with RNA or DNA, even if amino acid derivatives are used as backbones^26,29–31,34,35^, indicating that ribose is not necessary for formation of stable duplex structure. These findings raise up the fundamental question why nature selected ribose as backbone of genetic materials and amino acid as backbone of proteins, products of gene. It is also possible that acyclic nucleic acid, capable of hybridizing with RNA, derived from amino acid derivatives similar to our SNA and L-*a*TNA served as evolutionary intermediates or competitors of genetic material. To answer this, it might be important to consider the optimal helical structure for compact packing of large genetic polymer.

Thus, the structural data reported here will expand the scope of application of acyclic nucleic acid analogues in prebiotic studies as well as in nucleic acid-based drug and nanotechnology.

## Methods

### Oligonucleotide preparation and crystallization of L-*a*TNA-RNA and SNA-RNA duplexes

The oligomers used in this study, S8a: (*S*)-GCAGCAGC-(*R*), S7b: (*S*)-GCTGCTG-(*R*), _L_T8a: 3′-GCAGCAGC -1′, _L_T7b: 3′- GCTGCTG-1′, R8Br:5′ GCUGC^Br^UGC-3′, R8a: 5′-GCAGCAGC-3′, and R7b:5′-GCUGCUG-3′, were obtained from HSS Co., Ltd.. Purified oligomers were dissolved in 10 mM Tris-HCl (pH 7.0) and then duplexes were prepared at a final concentration of 1 mM. S8a/R8Br and _L_T8a/R8Br were annealed by heating for 10 min at 95 °C and then gradually cooling to 4 °C. Crystallization conditions were screened with commercially available sparse matrix screening kits. Crystals of S8a/R8Br were grown using the sitting drop method in 0.1 M HEPES (pH 6.5), 75 mM CaCl_2_, and 28% PEG400, and crystals of _L_T8a/R8Br were grown using the sitting drop method in 0.1 M HEPES (pH 7.5), 200 mM CaCl_2_, and 28% PEG400 at 20 °C.

### X-ray data collection and refinement

X-ray datasets were collected on the BL44XU beamline at SPring-8, Japan. X-ray diffraction datasets were integrated and scaled using XDS^61^ and AIMLESS^62^. The crystal structures were solved using the multi-wavelength anomalous dispersion method relying on the Br atoms in the RNA. The initial phases were determined with the Phenix AutoSol^63^. The obtained electron density maps were very clear, and the initial coordinates were built manually using COOT^64^. Model refinement was conducted using REFMAC5^65^ and phenix.refine^63^. Topology files for the model refinement of nucleoside moieities of L-*a*TNA and SNA monomers were created by PRODRG2 Server^66^. For _L_T8a/R8Br crystals, merohedral twinning was suspected during refinement because the refined coordinates had an *R*_work_ of 36% and an *R*_free_ of 40% in the *C*2 space group. Therefore, the structure belonging to the *I*2 space group was solved by molecular replacement methods using the *C*2 structure as a search model with MOLREP^67^, giving rise to a dramatically improved the refinement statistics with *R*_work_ of 26.9% and *R*_free_ of 28.8%. The crystallographic parameters and final refinement statistics of _L_T8a/R8Br and S8a/R8Br are summarized in Tables 1 and 2, respectively.

### Thermal melting analyses and CD measurement

L-*a*TNA, SNA and/or RNA (2.5 μM) were dissolved in 10 mM HEPES buffer (pH 7.4) with 100 mM CaCl_2_. The melting curves were obtained with a Shimazu UV-1800 by measuring the change in absorbance at 260 nm versus temperature. Melting curves were measured with a temperature ramp of 0.5 °C min^−1^. *T*_m_ values were determined from the maximum in the first derivative of the melting curve. For CD, L-*a*TNA, SNA, and/or RNA (4.0 μM) were dissolved in 10 mM HEPES buffer (pH 7.4) with 100 mM CaCl_2_. Spectra were recorded at 5 °C using a JASCO model J-820 instrument.

### Mass spectroscopy analysis under non-denaturing conditions

The oligonucleotides mixtures were buffer-exchanged into 100 mM triethylammonium acetate, pH 7.0, by passing the oligonucleotides through Bio-Rad Micro Bio-Spin 6 columns. After buffer exchange, samples were immediately analysed by nanoflow electrospray ionization mass spectrometry using gold-coated glass capillaries made in house (~3 μL sample loaded per analysis). Spectra were recorded on a Waters SYNAPT G2-Si HDMS mass spectrometer in negative ionization mode at 1.13 kV with 150 V sampling cone voltage and source offset voltage, 4 V trap and 2 V transfer collision energies, and 5 mL/min trap gas flow. The spectra were calibrated using 2 mg/mL cesium iodide dissolved in 50% 2-propanol and analysed using MassLynx software (Waters).

Calculated masses (singly charged states) are as follows: _L_T8a, 2650.7; R8Br, 2578.3; _L_T8a/R8Br duplex, 5228.9; dimer of _L_T8a/R8Br duplex, 10457.9; S8a, 2538.5; R8Br, 2578.3; S8a/S8a duplex, 5077.1; S8a/R8Br duplex, 5116.8; dimer of S8a/R8Br duplex, 10233.6; _L_T7b, 2314.6; _L_T8a/_L_T7b duplex, 4965.2; dimer of _L_T8a/_L_T7b duplex, 9930.5; S7b, 2216.5; S8a/S7b duplex, 4755.0; dimer of S8a/S7b duplex, 9510.0; S7b/S7b duplex, 4432.9.

## Supplementary information

Supplementary Information

## Data Availability

Structural data that support the findings of this study have been deposited in PDB with the accession code, 7BPF and 7BPG. All data analysis results generated during this study are included in this published article and its supplementary information file.
