# Supplementary material for: Intrastrand backbone-nucleobase interactions stabilize unwound right-handed helical structures of heteroduplexes of L-aTNA/RNA and SNA/RNA
Source: Commun Chem. 2020 Nov 6;3:156. doi: 10.1038/s42004-020-00400-2 (PMC9814321; doi:10.1038/s42004-020-00400-2)
Supplement: Supplementary file 1 — Supplementary Information [file 42004_2020_400_MOESM1_ESM.pdf]

Supplementary Information

Intrastrand backbone-nucleobase interactions stabilize unwound right-handed helical structures of heteroduplexes of L-*a*TNA/RNA and SNA/RNA

Yukiko Kamiya*,[1] Tadashi Satoh,[2] Atsuji Kodama,[3] Tatsuya Suzuki,[2,3,4] Keiji Murayama,[1] Hiromu Kashida,[1] Susumu Uchiyama,[3,5] Koichi Kato,[2,3,4] and Hiroyuki Asanuma*[1]

1. Graduate School of Engineering, Nagoya University, Furo-cho, Chikusa-ku, Nagoya 464-8603, Japan.
2. Graduate School of Pharmaceutical Sciences, Nagoya City University, 3-1 Tanabe-dori, Mizuho-ku, Nagoya 467-8603, Japan.
3. Exploratory Research Center on Life and Living Systems (ExCELLS), National Institutes of Natural Sciences, 5-1 Higashiyama, Myodaiji, Okazaki 444-8787, Japan.
4. Institute for Molecular Science, National Institutes of Natural Sciences, 5-1 Higashiyama, Myodaiji, Okazaki, 444-8787, Japan.
5. Graduate School of Engineering, Osaka University, Suita, Osaka 565-0871, Japan.

Supplementary Figures 1 – 8・・・・・・・・S2-S7

Supplementary Tables 1 – 5・・・・・・・・S8-S13

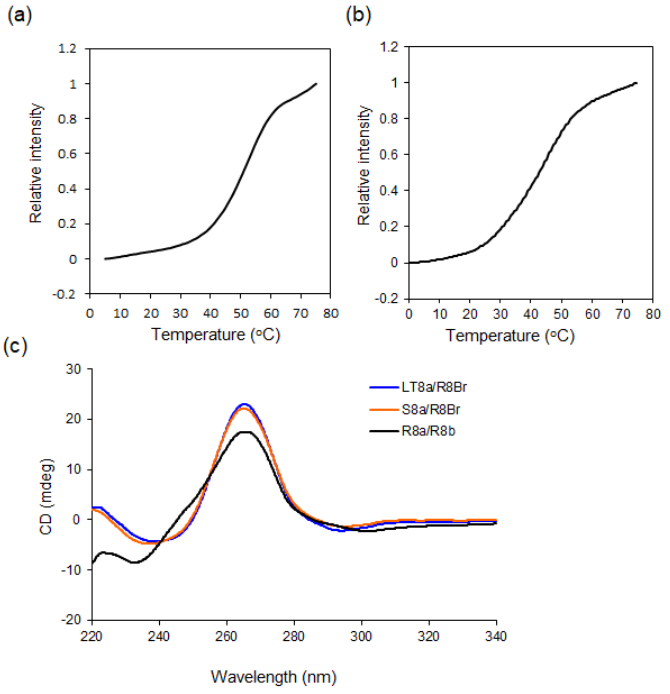

Supplementary Figure 1. Melting curves and CD spectra of L-*a*TNA/RNA, and SNA/RNA, and RNA/RNA duplexes.

Absorbance versus temperature curves for $_{\text{L}}$T8a/R8Br (a) and S8a/R8Br (b). Solution conditions were 10 mM HEPES (pH 7.4), 100 mM $CaCl_2$, and 2.5 μM oligonucleotide. (c) CD spectra of $_{\text{L}}$T8a/R8Br, S8a/R8Br, and R8a/R8b recorded at 5 ºC. Solution conditions for CD measurements were 10 mM HEPES (pH 7.4), 100 mM $CaCl_2$, 4.0 μM oligonucleotide.

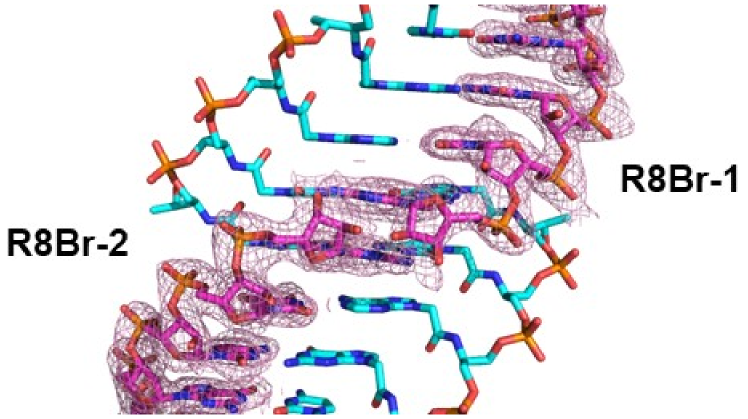

Supplementary Figure 2. Electron densities of 3' terminal C8 of RNA strands were not observed in L-*a*TNA/RNA

In the stick representations, carbon atoms are coloured magenta and cyan. N, O, and P atoms are coloured as in Fig. 1. Contours represent the $2F_o$-F_c electron density map at the 1.0 σ level.

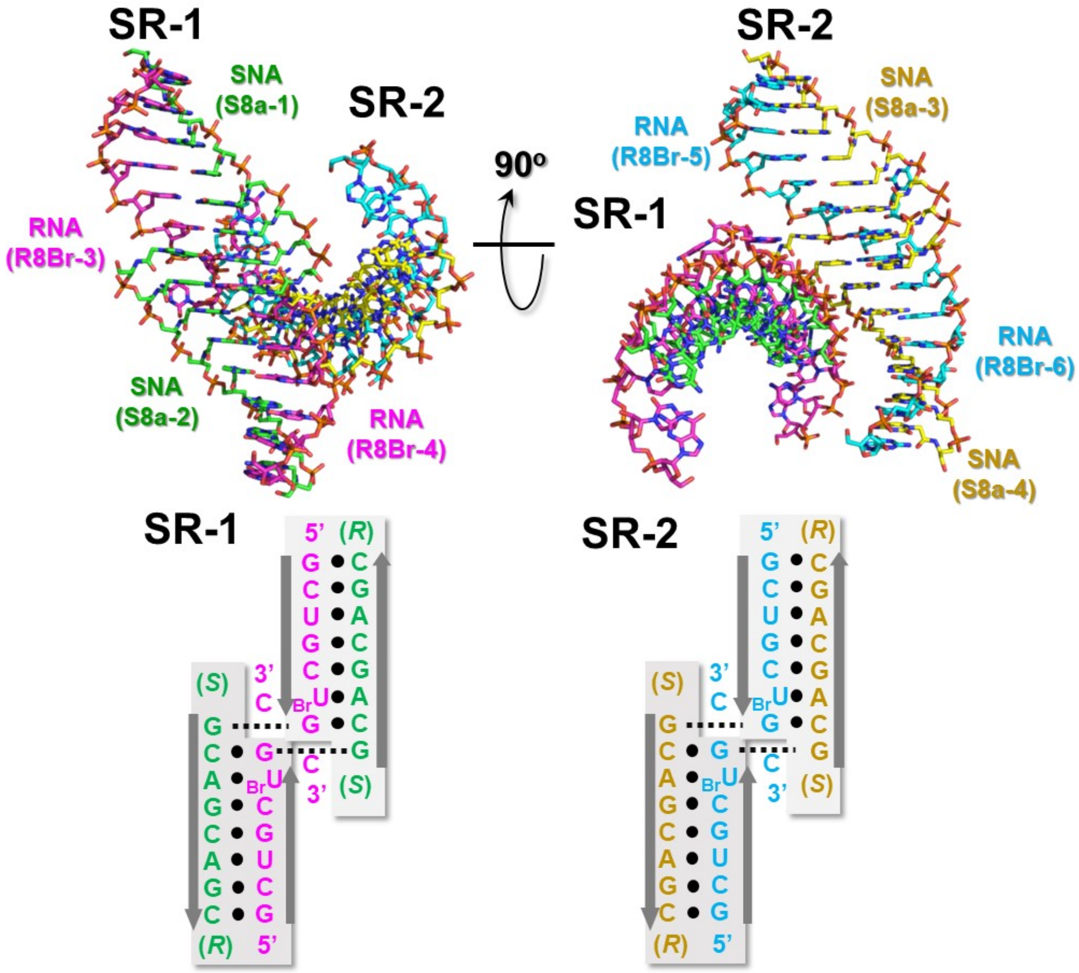

Supplementary Figure 3. SNA-RNA complexes observed in the asymmetric units in the crystal structure of S8a/R8Br.

In the stick representations, carbon atoms in RNAs (R8Br-3 and R8Br-4), RNAs (R8Br-5 and R8Br-6), SNAs (S8a-1 and S8a-2), and SNAs (S8a-3 and S8a-4) are coloured in magenta, cyan, green, and yellow, respectively. N, O, and P atoms are coloured as in Fig. 1. The base-pairing patterns in the asymmetric units are shown below the structures. Watson-Crick and Hoogsteen base pairs are indicated as black circles and dashed lines, respectively.

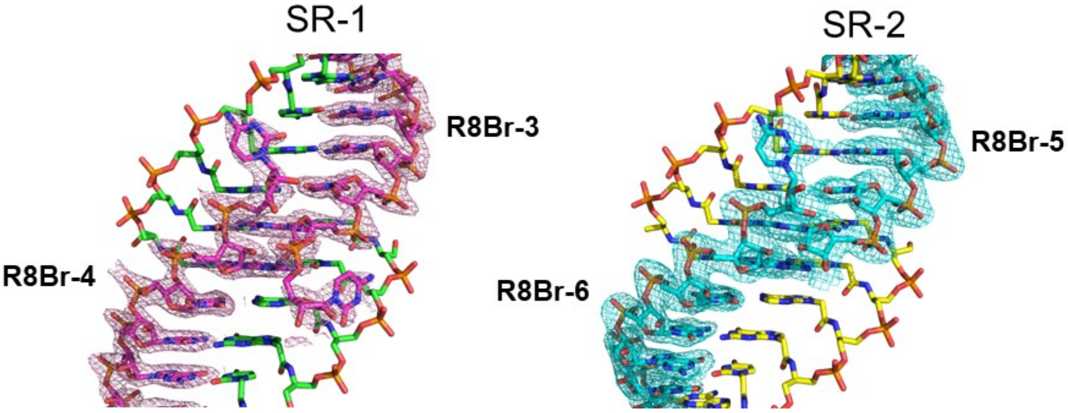

Supplementary Figure 4. Electron densities of 3' terminal regions of the RNA strands in SNA/RNA.

In the stick renderings, atoms are coloured magenta, green, cyan, and yellow for carbon. N, O, and P atoms are coloured as in Fig. 1. Contours represent the $2F_o$-F_c electron density map at the 1.0 σ level.

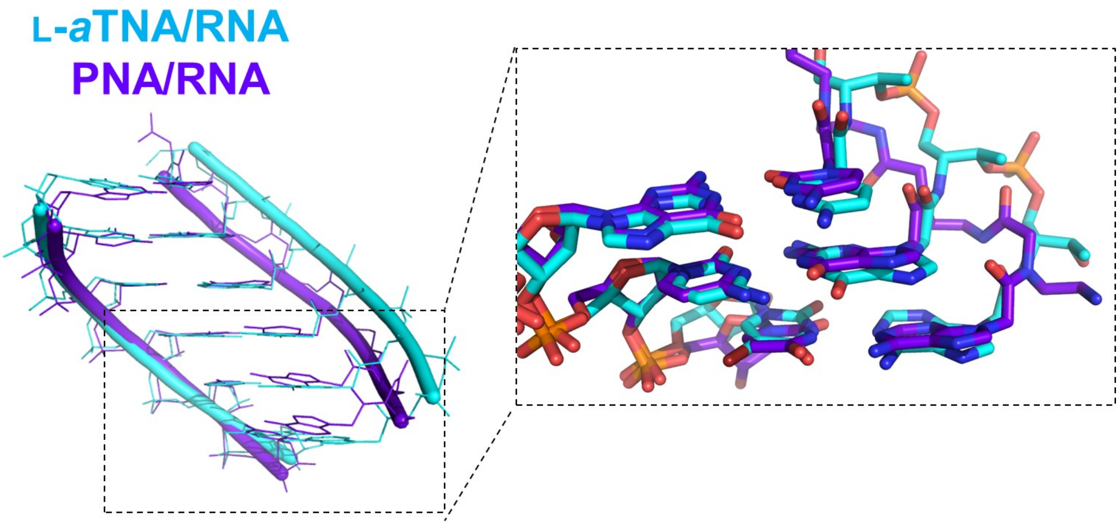

Supplementary Figure 5. Close up views of superposition of duplex structures of L-*a*TNA/RNA (cyan) and PNA/RNA (purple).

In the stick renderings, atoms are coloured cyan and purple for carbon N, O, and P atoms are coloured as in Fig. 1.

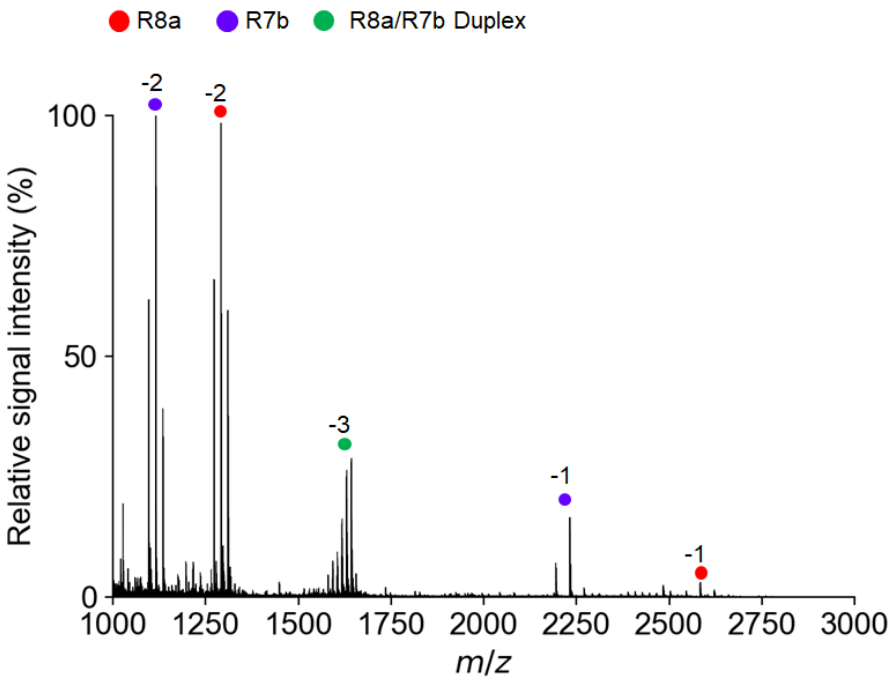

Supplementary Figure 6. nanoESI-MS analysis of mixture of R8a and R7b.

Mass spectrum of solution of R8a and R7b collected under non-denaturing conditions in negative ionization mode. Calculated masses (singly charged states) are as follows: R8a (red), 2650.7; R7b (purple), 2195.3; and R8a/R7b duplex (green), 4741.7.

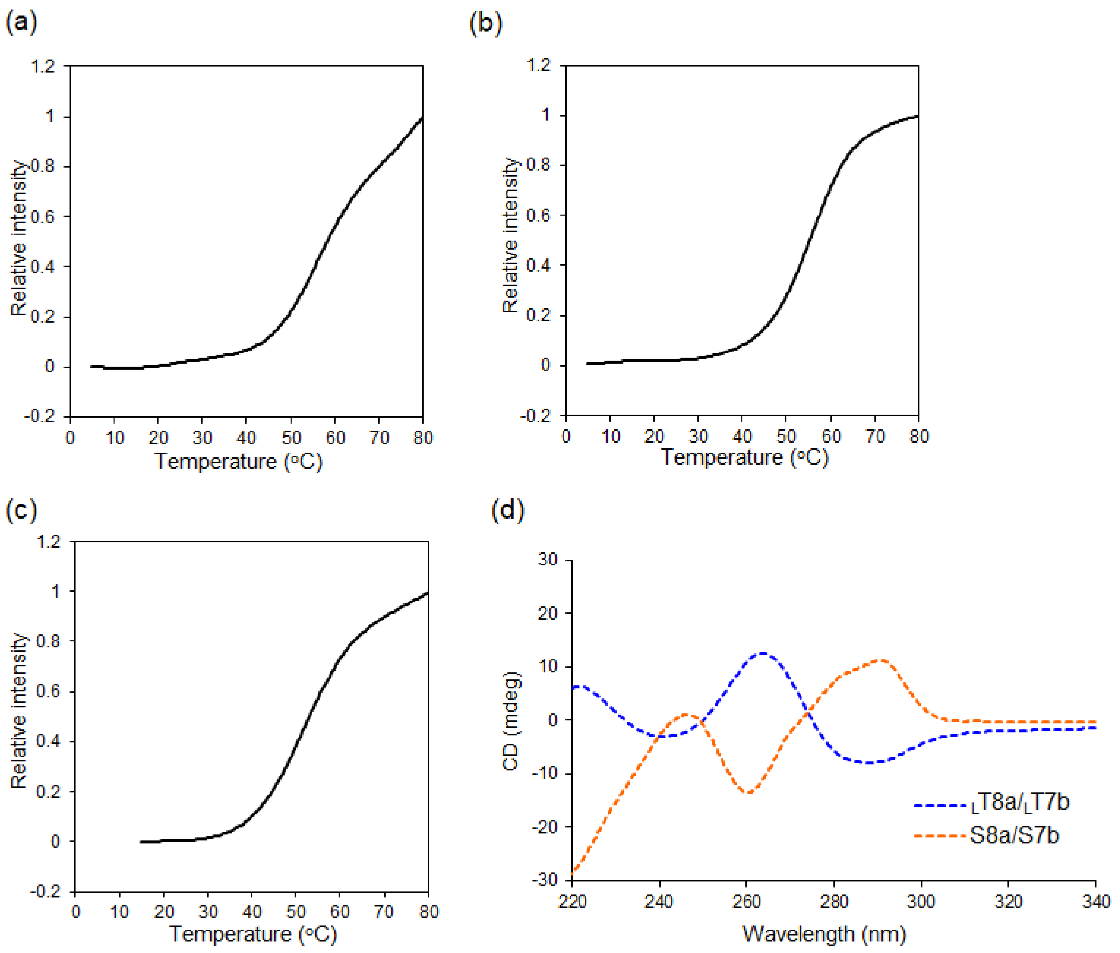

Supplementary Figure 7. Melting curves and CD spectra of homo duplexes of L-*a*TNA, SNA, and RNA.

Absorbance versus temperature curves for $_L$T8a/$_L$T7b (a), S8a/S7b (b), and R8a/R7b (c). Solution conditions were 10 mM HEPES (pH 7.4), 100 mM $CaCl_2$, and 2.5 µM oligonucleotide. (d) CD spectra of solution of $_L$T8a/$_L$T7b and S8a/S7b recorded at 5 °C. Solution conditions were 10 mM HEPES (pH 7.4), 100 mM $CaCl_2$, and 4.0 µM oligonucleotide.

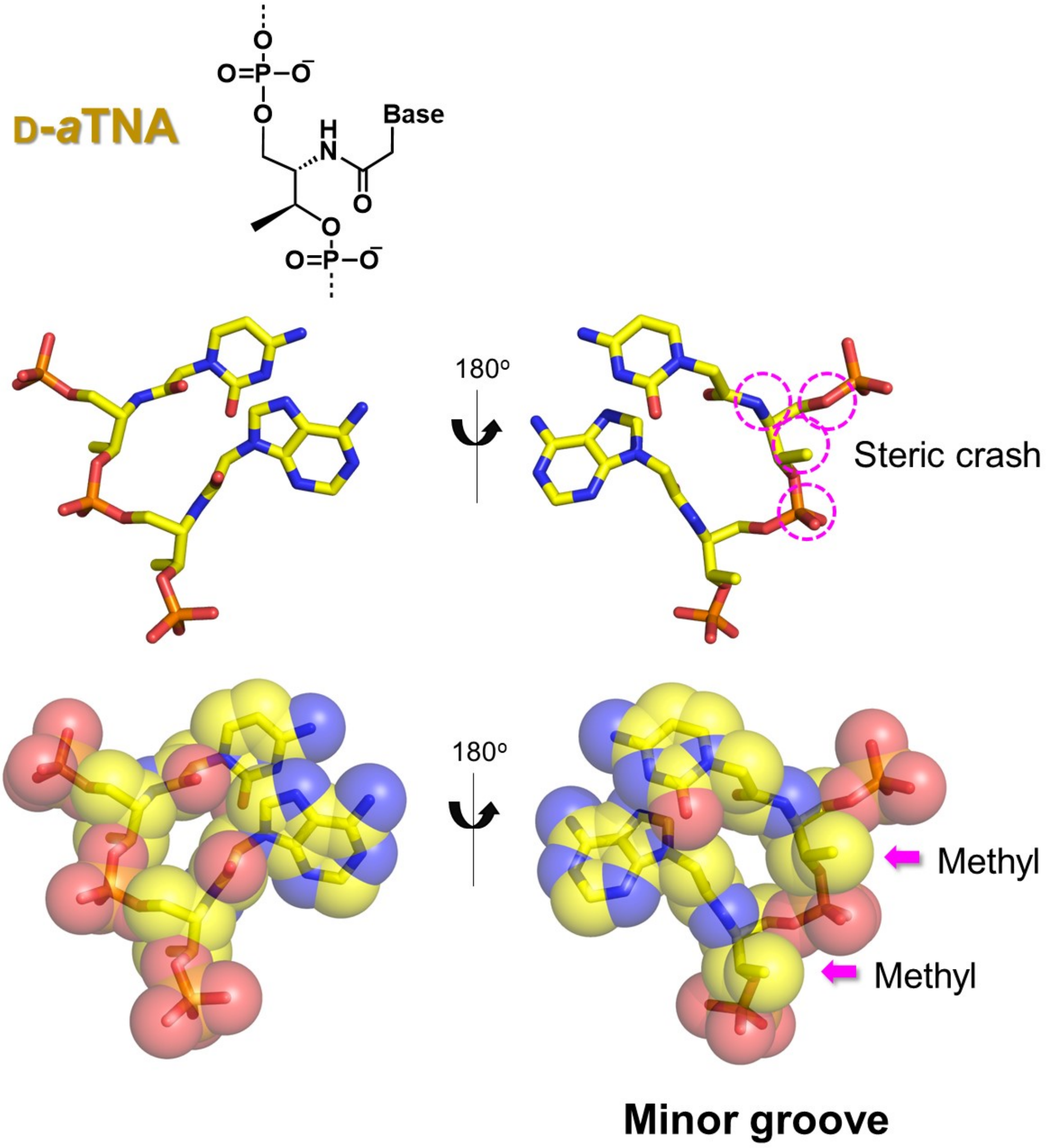

Supplementary Figure 8. Steric crash between methyl group of D-*a*TNA and oxygens of neighbouring phosphate group in right-handed helix structure model.

D-*a*TNA structure model was produced by attachment of methyl group at C1' position of SNA crystal structure. Methyl group is located in the minor groove. D-*a*TNA backbone structure is shown as stick and sphere representation. Protons are omitted. C is coloured in yellow. N, O, and P atoms are coloured as in Fig. 1.

Supplementary Table 1. Helical parameters of L-*a*TNA/RNA.[a]

L-*a*TNA/RNA[b]	X-displacement [Å]	Inclination [deg]	Helical rise [Å]	Helical twist [deg][c]	Tilt [deg][c]	Slide [Å]	Roll [deg][c]
GC/gc	-4.8	7.6	3.0	31.7	10.7	-2.2	4.1
CU/ag	-7.3	15.7	2.5	23.7	-0.2	-2.2	6.4
UG/ca	-6.4	5.2	3.0	21.8	0.3	-2.2	2.0
GC/gc	-8.2	0.2	3.1	18.7	2.3	-2.7	0.1
$C_{Br}U$/ag	-5.8	-5.4	3.8	20.9	-3.3	-2.5	-1.9
$_{Br}UG$/ca	-3.5	-7.3	3.3	26.1	1.8	-2.0	-3.3
ca/$_{Br}UG$	-3.5	-4.1	3.0	24.9	-3.1	-1.7	-1.8
ag/$C_{Br}U$	-5.6	-2.2	3.6	22.3	1.4	-2.3	-1.9
gc/GC	-10.8	9.6	2.5	16.3	-0.3	-2.6	2.7
ca/UG	-5.8	-0.6	3.1	23.4	-4.3	-2.4	-0.3
ag/CU	-9.1	-7.2	3.7	16.0	0.3	-2.9	-2.0
gc/GC	-6.3	7.3	2.9	27.5	-2.5	-2.6	3.5

[a]The duplex regions are highlighted in light blue background. All data were calculated using 3DNA-Web. [b]Capitals indicate RNA and small letters indicate L-*a*TNA. [c] Local base-pair step parameters.

Supplementary Table 2. Helical parameters of SNA/RNA duplex. [a]

SNA/RNA[b]	X-displacement [Å]	Inclination [deg]	Helical rise [Å]	Helical twist [deg]	Tilt [deg] [c]	Slide [Å] [c]	Roll [deg] [c]
**SR-1**							
GC/gc	-2.9	-6.1	3.4	30.6	-2.1	-1.9	-3.2
CU/ag	-5.6	-2.8	3.1	20.2	-5.4	-2.1	-1.0
UG/ca	-10.8	29.9	2.1	18.2	-0.5	-1.9	9.0
GC/gc	-6.8	1.5	3.2	22.3	-0.9	-2.6	0.6
$C_{br}U$/ag	-5.8	1.1	3.5	22.0	1.0	-2.2	0.4
$_{br}UG$/ca	-3.5	-3.8	3.0	24.3	-3.3	-1.7	-1.6
ca/$_{br}UG$	-3.3	-2.2	3.0	28.8	2.9	-1.8	-1.1
ag/$C_{br}U$	-6.2	-7.3	3.8	17.9	-1.0	-2.4	-2.3
gc/GC	-6.5	3.6	3.1	23.2	3.7	-2.4	1.4
ca/CG	-8.9	-17.5	3.6	11.6	3.5	-2.9	-3.4
ag/CC	-5.2	17.1	2.8	32.0	1.0	-1.9	9.3
gc/GC	-2.5	-8.1	3.4	33.7	1.7	-1.9	-4.7
**SR-2**							
GC/gc	-5.2	2.0	3.4	30.5	4.8	-2.6	1.0
CU/ag	-7.3	6.6	2.8	21.4	-1.5	-2.3	2.5
UG/ca	-4.6	-5.5	3.6	20.9	3.7	-2.0	-2.0
GC/gc	-7.5	9.8	3.1	22.0	3.3	-2.4	3.7
$C_{br}U$/ag	-7.8	5.1	3.8	20.0	-3.1	-2.4	1.8
$_{br}UG$/ca	-3.8	-5.9	3.0	25.5	2.8	-2.0	-2.6
ca/$_{br}UG$	-3.6	-5.4	3.3	25.7	-4.3	-1.9	-2.4
ag/$C_{br}U$	-4.7	-6.5	3.5	23.3	2.2	-2.3	-2.6
gc/GC	-8.4	5.1	3.1	20.2	-2.5	-2.7	1.8
ca/UG	-5.8	2.2	2.1	20.9	-4.1	-2.0	0.8
ag/CU	-7.8	18.8	3.1	22.5	1.9	-2.1	7.2
gc/GC	-2.0	-2.5	3.4	44.1	-2.4	-1.6	-1.9

[a] The duplex regions are highlighted in light blue background. All data were calculated using 3DNA-Web. [b] Capitals indicate RNA and small letters indicate SNA. [c] Local base pair step parameters.

Supplementary Table 3. Torsion angles (deg.) in L-*a*TNA/RNA hetero duplexes.

		α	β	γ	δ	ε	ζ	χ
R8Br-1[a]	G1	---	---	15.0	92.2	-153.9	-62.2	-174.9
	C2	-122.4	-168.1	91.1	72.4	-141.1	-81.8	-168.8
	U3	-45.9	167.8	48.3	76.4	-161.4	-71.1	-162.9
	G4	-73.4	178.8	59.7	75.7	-160.8	-62.6	-165.4
	C5	-103.4	165.2	103.1	69.8	-148.4	-85.1	-177.3
	$_{Br}$U6	-49.2	159.0	58.1	79.1	-167.3	-77.7	-172.8
	G7	-77.4	-164.1	58.4	90.3	---	---	-149.7
R8Br-2[a]	G1	---	---	-81.0	93.4	-142.9	-67.1	-178.9
	C2	-79.5	-177.2	60.6	74.6	-156.4	-76.5	-174.2
	U3	-66.7	172.8	75.4	78.2	-157.5	-78.6	-174.2
	G4	-73.2	179.5	61.3	72.1	-151.8	-75.5	-167.1
	C5	-70.5	161.7	79.9	75.5	-155.9	-79.8	-173.9
	$_{Br}$U6	-69.3	173.2	65.4	81.1	-147.7	-89.5	-166.5
	G7	-66.7	169.7	56.0	77.3	---	---	-154.8

		α	β	γ	δ	ε	ζ
$_{L}$T8a-1[b]	G1	---	---	117.6	-62.5	-105.1	50.8
	C2	90.5	177.3	-62.7	-68.2	-149.7	66.8
	A3	85.9	179.7	-57.2	-71.6	-135.2	68.3
	G4	86.0	172.6	-54.8	-59.1	-135.6	48.9
	C5	92.5	170.0	-53.3	-63.3	-134.0	46.8
	A6	89.7	173.6	-53.7	-68.7	-142.1	68.8
	G7	78.0	174.8	-48.8	-76.5	-142.5	81.5
	C8	86.7	170.3	-68.5	137.3	---	---
$_{L}$T8a-2[b]	G1	---	---	106.8	-57.2	-167.6	53.9
	C2	88.0	172.7	-64.5	-62.6	-150.8	63.5
	A3	76.7	-179.0	-57.8	-63.9	-128.6	45.4
	G4	92.8	171.9	-56.9	-61.2	-126.8	53.5
	C5	103.9	158.0	-56.8	-59.9	-140.1	48.9
	A6	79.9	179.0	-51.7	-60.3	-132.1	54.9
	G7	88.9	165.2	-52.3	-62.3	-140.9	62.3
	C8	93.1	170.3	-71.8	128.0	---	---

[a]All values were calculated using 3DNA-Web. [b]All values were measured using pymol.

Supplementary Table 4. Torsion angles (deg.) of SNA/RNA hetero duplexes.

		α	β	γ	δ	ε	ζ	χ
R8Br-3[a]	G1	---	---	71.6	81.1	-140.2	-78.6	176.0
	C2	-83.6	173.5	70.4	80.8	-144.6	-80.2	-168.4
	U3	-60.2	166.0	58.2	70.8	-127.7	-87.9	-156.5
	G4	-85.5	141.7	92.7	70.3	-150.6	-79.5	-176.2
	C5	-56.0	165.2	64.1	75.0	-156.3	-74.7	-171.1
	$_{Br}$U6	-74.5	169.9	73.9	83.0	-158.5	-87.4	-164.7
	G7	-63.9	174.3	72.4	141.7	-94.4	-69.0	-136.1
R8Br-4[a]	G1	---	---	-79.7	109.8	-133.9	8.7	178.5
	C2	-135.1	73.5	168.7	96.2	-105.6	-91.2	175.3
	U3	-59.3	165.7	31.2	75.6	-150.5	-41.4	-160.2
	G4	-128.3	110.7	157.7	82.1	-139.4	-79.3	-177.5
	C5	-54.5	172.5	51.5	74.8	-177.5	-84.9	-165.3
	$_{Br}$U6	162.1	-151.4	175.0	93.0	-121.9	-86.6	176.2
	G7	-74.5	167.0	56.8	78.0	-154.2	-123.2	-162.0
R8Br-5[a]	G1	---	---	8.9	83.4	148.3	39.2	-176.7
	C2	147.1	132.0	151.7	87.4	-143.1	-75.6	-173.4
	U3	-67.7	179.2	56.4	80.1	-160.1	-78.9	-167.2
	G4	-72.9	173.9	62.8	76.9	-157.3	-70.7	-163.8
	C5	-83.0	169.6	82.6	74.4	-152.7	-84.1	-171.5
	$_{Br}$U6	-64.8	166.0	67.5	79.0	-153.1	-83.5	-172.4
	G7	-60.7	177.3	50.0	79.7	-152.5	-117.1	-159.2
R8Br-6[a]	G1	---	---	-93.1	99.3	-133.8	-74	-179.5
	C2	-58.5	171.5	45.8	79.1	-138.8	-85.1	-167.2
	U3	-50.3	168.5	45.3	74.1	-155.8	-74.6	-165.1
	G4	-61.7	167.9	62.4	74.4	-166.7	-70.4	-162.9
	C5	144.7	-171.1	-170	83.2	-151.6	-70.5	-176.9
	$_{Br}$U6	-78.2	174.7	69.5	82.7	-155.6	-85.6	-170.9
	G7	-67.0	177.9	54.2	81.2	---	---	-153.8

		α	β	γ	δ	ε	ζ
S8a-1[b]	G1	---	---	-55.2	-58.7	-161.3	67.2
	C2	88.2	-168.2	-68.2	-50.3	-147.6	64.3
	A3	75.4	-168.2	-65.4	-53.9	-158.7	60.9
	G4	70.6	-164.8	-50.2	-70.3	-137.7	76.7
	C5	74.1	-177.0	-54.0	-55.1	-157.0	45.8
	A6	132.7	173.1	-64.5	-58.3	-143.9	65.4
	G7	86.4	-173.0	-56.4	-52.1	-156.7	68.1

	C8	82.4	-163.3	-73.8	35.8	---	---
S8a-2[b]	G1			-116.9	-53.3	-178.7	59.2
	C2	83.9	174.9	-65.8	-66.7	-142.1	73.0
	A3	70.7	-175.7	-57.5	-61.9	-151.1	61.7
	G4	68.5	-176.6	-52.8	-65.7	-157.2	62.5
	C5	79.8	-173.8	-57.9	-56.3	-163.9	74.7
	A6	54.9	-161.2	-51.5	-58.2	-140.1	79.2
	G7	80.9	178.1	-60.1	-64.5	-160.2	73.8
	C8	90.4	-82.4	158.8	-160.1	---	---
S8a-3[b]	G1			174.5	-63.9	158.1	64.8
	C2	70.6	-160.6	-61.8	-70.8	-169.9	73.3
	A3	68.6	-162.9	-58.1	-61.5	-139.1	73.1
	G4	68.4	-175.3	-55.8	-62.5	-161.1	68.0
	C5	72.7	-173.3	-58.3	-56.6	-144.8	63.2
	A6	89.4	176.4	-65.4	-65.5	-157.1	78.3
	G7	67.2	-167.0	-49.2	-68.1	-129.8	69.8
	C8	-154.2	-177.9	-179.7	-67.6	---	---
S8a-4[b]	G1			168.7	-50.8	-170.6	26.5
	C2	89.6	-168.5	-58.5	-54.9	-155.2	70.8
	A3	67.2	-162.6	-59.5	-55.8	-155.6	60.7
	G4	81.6	-170.8	-64.1	-57.8	-140.4	73.8
	C5	77.9	173.3	-58.2	-63.2	-155.4	81.7
	A6	58.7	-164.7	-51.5	-61.4	-138.1	92.2
	G7	90.0	-175.1	-60.8	-54.9	-161.4	57.8
	C8	89.7	178.8	-52.7	-73.9	---	---

[a]All values were calculated using 3DNA-Web. [b]All values were measured using pymol.

Supplementary Table 5. C---O distances (Å) between backbone and neighboring nucleobase in L-*a*TNA and SNA of L-*a*TNA/RNA and SNA/RNA[a]

	L-*a*TNA in L-*a*TNA/RNA				SNA in SNA/RNA		
	**O5'-C8/C6**	**O5'-C6'**	**O2-C6'**		**O5'-C8/C6**	**O5'-C6'**	**O2-C6'**
**$_{L}$T8a-1**				**S8a-1**			
G1-C2	3.4	3.5	-	G1-C2	3.4	3.5	-
C2-A3	3.3	3.5	3.3	C2-A3	3.1	3.3	3.2
A3-G4	3.5	3.3	-	A3-G4	3.2	3.5	-
G4-C5	3.6	3.5	-	G4-C5	3.7	3.6	-
C5-A6	3.2	3.5	3.3	C5-A6	3.3	3.5	3.1
A6-G7	3.4	3.6	-	A6-G7	3.3	3.4	-
G7-C8	3.0	3.7	-	G7-C8	3.2	3.6	-
**$_{L}$T8a-2**				**S8a-2**			
G1-C2	3.6	3.5	-	G1-C2	3.5	3.6	-
C2-A3	3.2	3.4	3.1	C2-A3	3.2	3.4	3.3
A3-G4	3.5	3.4	-	A3-G4	3.5	3.6	-
G4-C5	4.0	3.4	-	G4-C5	3.3	3.4	-
C5-A6	3.2	3.2	3.4	C5-A6	3.3	3.6	3.3
A6-G7	3.8	3.4	-	A6-G7	3.2	3.6	-
G7-C8	3.2	4.0	-	G7-C8	3.0	4.8	-
				**S8a-3**			
				G1-C2	3.6	3.5	-
				C2-A3	3.2	3.5	3.2
				A3-G4	3.3	3.5	-
				G4-C5	3.5	3.4	-
				C5-A6	3.2	3.5	3.3
				A6-G7	3.4	3.2	-
				G7-C8	3.2	5.0	-
				**S8a-4**			
				G1-C2	4.0	3.9	-
				C2-A3	3.1	3.3	3.2
				A3-G4	3.6	3.2	-
				G4-C5	3.4	3.5	-
				C5-A6	3.1	3.4	3.4
				A6-G7	3.4	3.4	-
				G7-C8	3.5	3.6	-

[a]All values were measured using pymol.
